# SLC25A1, or CIC, is a novel transcriptional target of mutant p53 and a negative tumor prognostic marker

**DOI:** 10.18632/oncotarget.1831

**Published:** 2014-03-16

**Authors:** Vamsi K. Kolukula, Geetaram Sahu, Anton Wellstein, Olga C. Rodriguez, Anju Preet, Vito Iacobazzi, Gabriella D'Orazi, Chris Albanese, Ferdinando Palmieri, Maria Laura Avantaggiati

**Affiliations:** ^1^ Department of Oncology, Lombardi Comprehensive Cancer Center, Georgetown University, Washington, DC, USA; ^2^ Department of Biosciences, Biotechnology and Pharmacological Sciences, University of Bari, Italy; ^3^ Department of Experimental Oncology, Molecular Oncogenesis Laboratory, Regina Elena National Cancer Institute, Rome, Italy; ^4^ Department of Medical, Oral and Biotechnological Sciences, University of Chieti, Chieti, Italy

**Keywords:** SLC25A1, CIC, citrate, cancer, p53 mutations, mutant, FOXO-1, survival, prognostic, prognosis, marker

## Abstract

Mutations of the *p53* gene hallmark many human cancers. Several p53 mutant proteins acquire the capability to promote cancer progression and metastasis, a phenomenon defined as Gain of Oncogenic Function (GOF). The downstream targets by which GOF p53 mutants perturb cellular programs relevant to oncogenesis are only partially known. We have previously demonstrated that SLC25A1 (CIC) promotes tumorigenesis, while its inhibition blunts tumor growth. We now report that CIC is a direct transcriptional target of several p53 mutants. We identify a novel interaction between mutant p53 (mutp53) and the transcription factor FOXO-1 which is responsible for regulation of CIC expression levels. Tumor cells harboring mutp53 display higher CIC levels relative to p53 null or wild-type tumors, and inhibition of CIC activity blunts mutp53-driven tumor growth, partially overcoming GOF activity. CIC inhibition also enhances the chemotherapeutic potential of platinum-based agents. Finally, we found that elevated CIC levels predict poor survival outcome in tumors hallmarked by high frequency of p53 mutations. Our results identify CIC as a novel target of mutp53 and imply that the employment of CIC inhibitors may improve survival rates and reduce chemo-resistance in tumors harboring these types of mutations, which are among the most intractable forms of cancers.

## INTRODUCTION

In normal cells the p53 tumor suppressor restrains proliferation mostly by implementing a complex transcriptional network that in turn, promotes growth arrest, apoptosis or senescence in response to various forms of stress [1]. Missense mutations within the *p53* gene occur with high frequency in human tumors and are predominantly clustered within the DNA binding domain leading to loss of normal wild-type activity. However, it is emerging that p53 mutants also Gain novel Oncogenic Functions (GOF), thus explaining why one mutated copy of the p53 allele in the absence of a wild-type allele is often maintained even in genomic unstable, advanced forms of neoplasias [2,3].

The contribution of GOF mutants to tumorigenesis is illustrated by paradigmatic studies that have been conducted in mice and humans. Unlike p53 null animals that mainly develop soft tissue and hematopoietic tumors, mice expressing the “hot spot” p53 mutant proteins, p53R172H, p53R270H or p53R248W, display accelerated tumor onset and develop carcinomas in multiple tissues [4-8]. Furthermore, patients affected by Li-Fraumeni syndrome carrying germ-line *p53* GOF mutations develop more aggressive tumors and at an earlier age compared to patients lacking p53 or harboring loss of function mutations [7]. The downstream targets that mediate this pro-oncogenic activity of p53 mutants are complex and are only partially defined. It has been shown that the interaction of mutp53 with various transcription factors can positively or negatively regulate the expression of numerous target genes, in turn perturbing biological programs relevant to oncogenesis [3]. For example, the interaction of mutp53 with SREBP-1 or with NF-Y leads to alterations of the metabolism or of cell cycle checkpoints, respectively [9,10].

The mitochondrial citrate transporter SLC25A1, also known as CIC or CTP, belongs to a family of proteins embedded in the inner mitochondrial membrane and promotes the efflux of tricarboxylic citrate to the cytoplasm in exchange for dicarboxylic cytosolic malate [11-13]. Our previous work demonstrated that CIC expression is high in several tumor types and that its genetic or chemical inhibition has anti-tumor activity [14]. The relevance of CIC in cancer is further underlined by recent observations demonstrating that the transcription rates of the CIC promoter are positively regulated by key oncogenic molecules, specifically by PGC1α, by NK-

F-kappa-B and by inflammatory signals [15, 16]. Furthermore, CIC is also induced by Hepatitis C Virus, a major etiopathogenic factor for hepatocellular carcinoma [17].

In this work we asked whether regulation of CIC plays a role in mutp53 GOF activity, and we explored the molecular mechanisms underlying the cross-talk between CIC and mutp53 as well as the functional consequences of CIC inhibition in p53 mutant tumors. Our results demonstrate that several p53 mutants are directly recruited to the CIC promoter *via* a newly identified interaction with the transcription factor FOXO-1, resulting in induction of CIC transcription. Our studies identify CIC as a novel target of mutant, but not wild-type p53, thus offering new insights for understanding how p53 mutant proteins acquire oncogenic activity. Further, our results strongly argue that the inhibition of CIC may improve survival rates and chemo-resistance in tumors harboring p53 mutations.

## RESULTS

### Identification of CIC as a gene product regulated by several p53 mutants

**Figure 1:**
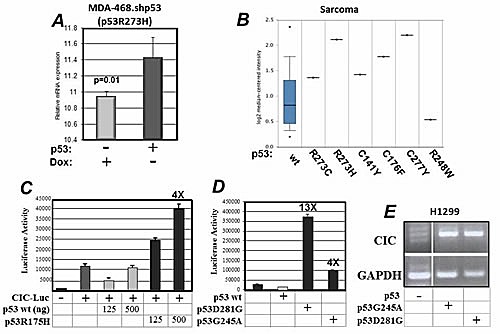
A. mRNA expression profiles derived from MDA-468shp53 cells grown in the presence or absence of doxycycline as described in [9,24] (tet-off). Data were extracted from the geoprofile database, plotted in an excel file and analyzed. B. Analysis of the Oncomine database for co-expression of CIC and p53 mutations. C. The p53 null H1299 lung cancer cells were non-transfected (−) or were transfected with the CIC-Luciferase vector (+) alone or with 120 or 500 ng of a vector expressing wild-type or p53R175H as indicated at the bottom of the panel. D. Similar luciferase experiments were performed by using two additional mutants p53G281D or p53G245A. E. Analysis of the CIC mRNA in H1299 cells, or in cells expressing p53G281 or p53G245A.

CIC was originally identified in micro-array platforms performed on the p53 null H1299 lung cancer cell line, expressing the “hot spot” mutant p53R175H or p53G245A. The analysis of existing databases, specifically either the geoprofiles [18,19] or Oncomine [20], further revealed that high CIC levels correlate with the expression of p53 mutations in several cancer cell lines or human tumors. Data extracted from the geoprofile database demonstrated that down-regulation of p53 with a specific shRNA in the breast cancer cell line MDA-468 that expresses p53R273H (MDA-468.shp53, [9]), reduces the CIC mRNA (Figure 1A). CIC expression is high in patient-derived osteosarcomas expressing some, but not all p53 mutations (Figure 1B). A similar association between p53 mutations and high CIC levels was confirmed by interrogating the cBioPortal database for cancer genomics [21,22] (Supplemental Figure S1).

To then determine whether wild-type or mutant p53 can regulate CIC at a transcriptional level, we performed luciferase assays using a vector containing the CIC promoter cloned upstream of the luciferase gene (PGL3-CIC) [23]. We found that while wild-type p53 repressed CIC promoter activity, p53R175H stimulated the CIC promoter in a dose-dependent fashion (Figure 1C). Two additional mutants, namely p53G281D and p53G245A were similarly able to activate the CIC reporter, although to different extents (Figure 1D). Furthermore, by employing *reverse transcription polymerase chain reaction* (RT-qPCR) we determined that both p53G281D and p53G245A enhanced CIC mRNA levels (Figure 1E), indicating that regulation of CIC occurs at a transcriptional level. To further substantiate our findings we also compared the expression levels of the CIC mRNA in tumors harboring p53 mutations, relative to the expression of other known, well validated targets of mutp53, specifically, cyclin A1 (CCN1) and cdk1 [10], as well as 3-Hydroxy-3-Methylglutaryl-Coenzyme A Reductase (HMGCR) and Mevalonate (Diphospho- Decarboxylase (MVD) [9]. As shown in Figure S2, the expression levels of CIC are found elevated in p53 mutant tumors with a frequency similar, if not greater, to those of these well validated targets.

**Figure 2:**
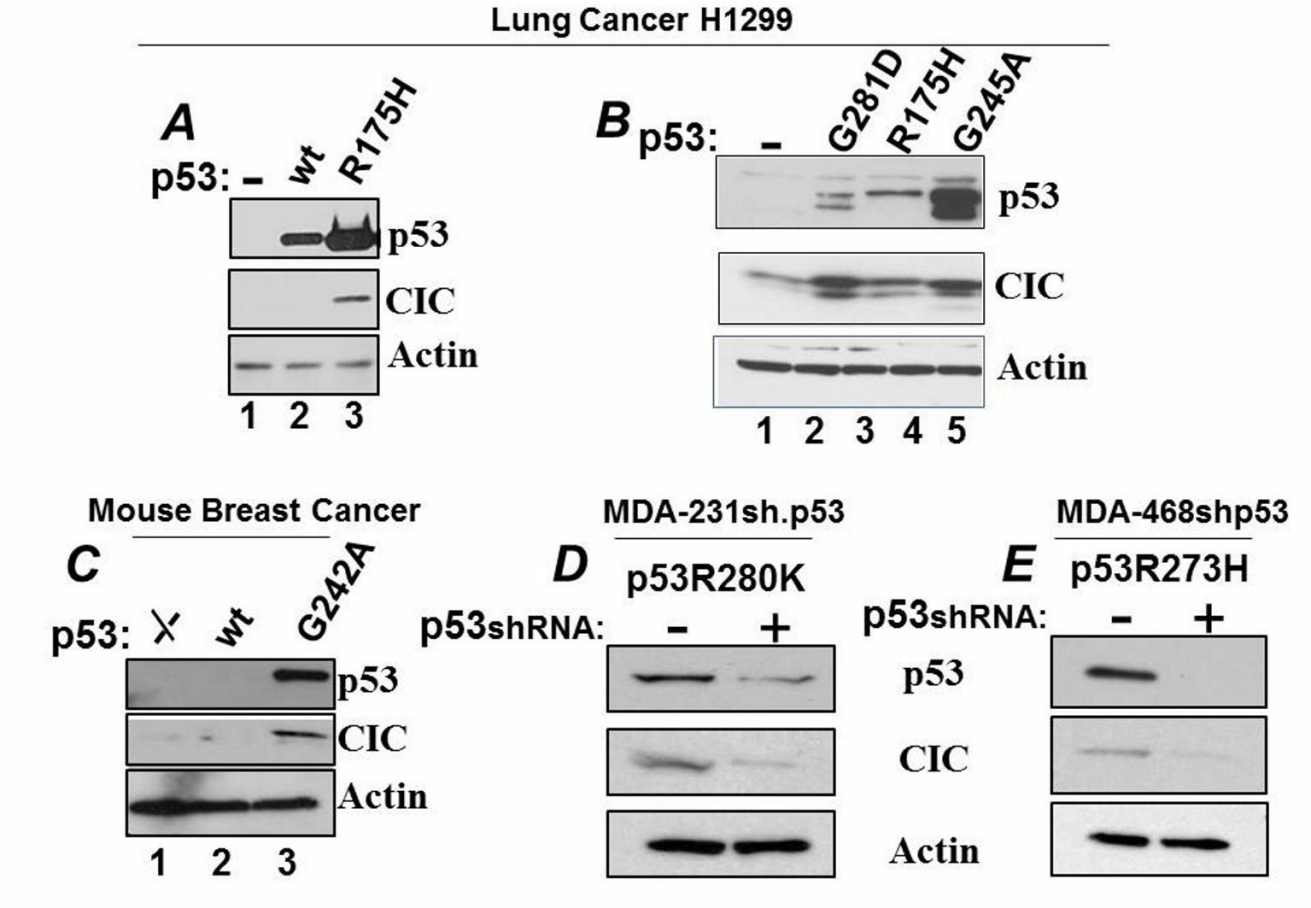
Expression of mutant p53 regulate CIC levels A. Lane 1, H1299 transfected with control vector; lane 2: transfection with the wt-p53 expressing vector; lane 3: transfection with the p53R175H vector. Cell lysates derived from these transfection experiments were subjected to immuno-blot with the anti-p53 (upper panel); with the anti-CIC (second panel), or with anti-actin antibodies (lower panel). B. CIC levels in H1299 cells or in H1299 cells stably expressing the p53 mutants indicated at the top of the panel. C. CIC expression levels in isogenic cell lines derived from murine mammary tumor cancers. Three cell lines were used, p53-/- (lane 1), p53 wild-type (lane 2) or cells harboring a mutation at position 245 that replaces a glycine with an alanine (p53A242G; lane 3). D-E. MDA-231 (D) or MDA-468 cells (E), harboring a tetracycline-inducible vector (tet-off) expressing the p53 shRNA were grown in the presence of doxycycline (p53shRNA off), or in its absence (p53shRNA on), cell extracts were prepared and analyzed as described before.

We next sought to confirm that p53 mutants increase the levels of CIC protein. The expression of p53R175H, p53G281D or p53G245A in the p53 null H1299 cell lines increased the levels of endogenous CIC protein compared to naive H1299 cells (Figure 2AB), while wild-type p53 did not (Figure 2A, lane 2). In isogenic murine mammary breast cancer cell lines either lacking p53, harboring a wild-type gene or carrying the p53G242A codon (equivalent to the human G245A mutation) [24-26], high CIC levels were seen in cells expressing mutant but not wild-type p53 (Figure 2C). Additionally, down-regulation of p53R280K or p53R273H in MDA-231 or MDA-468 cells, respectively, with the previously described specific p53shRNA [9,24] reduced the levels of endogenous CIC (Figure 2DE). These data demonstrate that a subset of p53 mutants enhance CIC expression.

### CIC expression is regulated by a newly identified interaction between mutant p53 and FOXO-1

**Figure 3:**
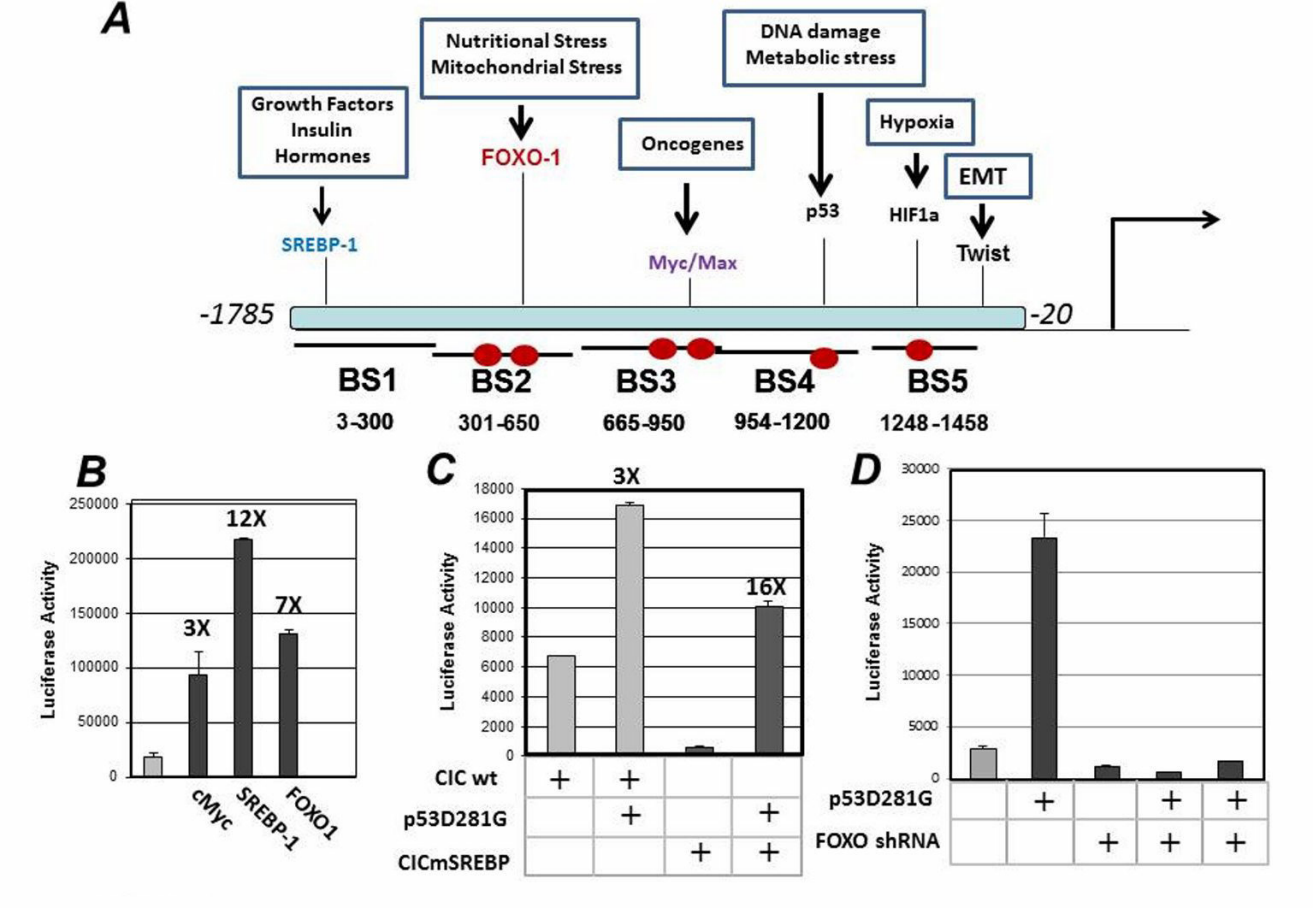
A. In silico analysis of the human CIC promoter. The CIC promoter regions spanning from -1785 to -20 were analyzed with Mathinspector (Genomatix) or with the LASAGNA software for *transcription factor* binding sites. Binding sites for various transcription factors and their biological significance are indicated. The promoter was divided in five fragments, indicated as Binding Sites 1-5. Red ovals indicate the position of the FOXO-1 binding elements (see also Supplemental Figure S3). B. Luciferase assays performed with the CIC-luciferase vector, in the absence or presence of SREBP-1, cMyc, FOXO-1 and PTEN. C. H1299 cells were transfected either with wild-type CIC luciferase reporter or with the mutant lacking the SREBP-1 binding element in the presence or absence of 250 ng or 500 ng of p53D281G. D. H1299 cells were transfected with control shRNA or with the shRNA specific for FOXO-1, in the presence or absence of the p53D281G expressing vector.

The molecular mechanisms by which p53 mutants activate transcription are only partially clear, but in many cases these proteins are directly recruited to various promoters via the interaction with other transcription factors [3]. To discriminate whether CIC is a direct or indirect target of mutp53, we first conducted an *in silico* analysis of the CIC promoter to detect *transcription factor* binding sites using Genomatix *MathInspector* and the LASAGNA search software (Figure 3A) [27]. In addition to the previously identified SREBP-1 binding site [23], these analyses revealed the presence of consensus elements for Myc/Max, for FOXO-1, for HIF1-alpha as well as for the transcription factor Twist, which plays a key role in the epithelial to mesenchymal transition (EMT) (Figure 3A). Interestingly, the CIC promoter also contains consensus sites for p53 as well, but given that wild-type p53 was unable to stimulate CIC promoter activity, the physiological relevance of these sites is currently unclear. It is possible that these sites are employed by p53 family members, p63 or p73. As previously shown by Infantino et al., [23], expression of SREBP-1 strongly stimulated CIC transcription and we further determined that also c-Myc and FOXO-1 induced CIC promoter activity (Figure 3B). The structure of the CIC promoter thus provides the potential for regulation of CIC expression levels by various oncogenic and anti-oncogenic signal pathways.

Recent studies have shown that various p53 mutants interact with SREBP-1 to induce the expression of SREBP-1 regulated genes [9]. Therefore, we next explored whether SREBP-1 influences the ability of mutant p53 to activate the CIC promoter. Surprisingly, both the p53D281G (Figure 3C) and p53R175H (not shown) mutants stimulated the transcription of the CIC promoter construct that lacked the SREBP-1 binding site [23] even more potently than the native promoter containing this element (Figure 3C). In contrast, the expression of a validated shRNA for FOXO-1, abrogated the ability of p53D281G to stimulate CIC promoter activity (Figure 3D).

**Figure 4:**
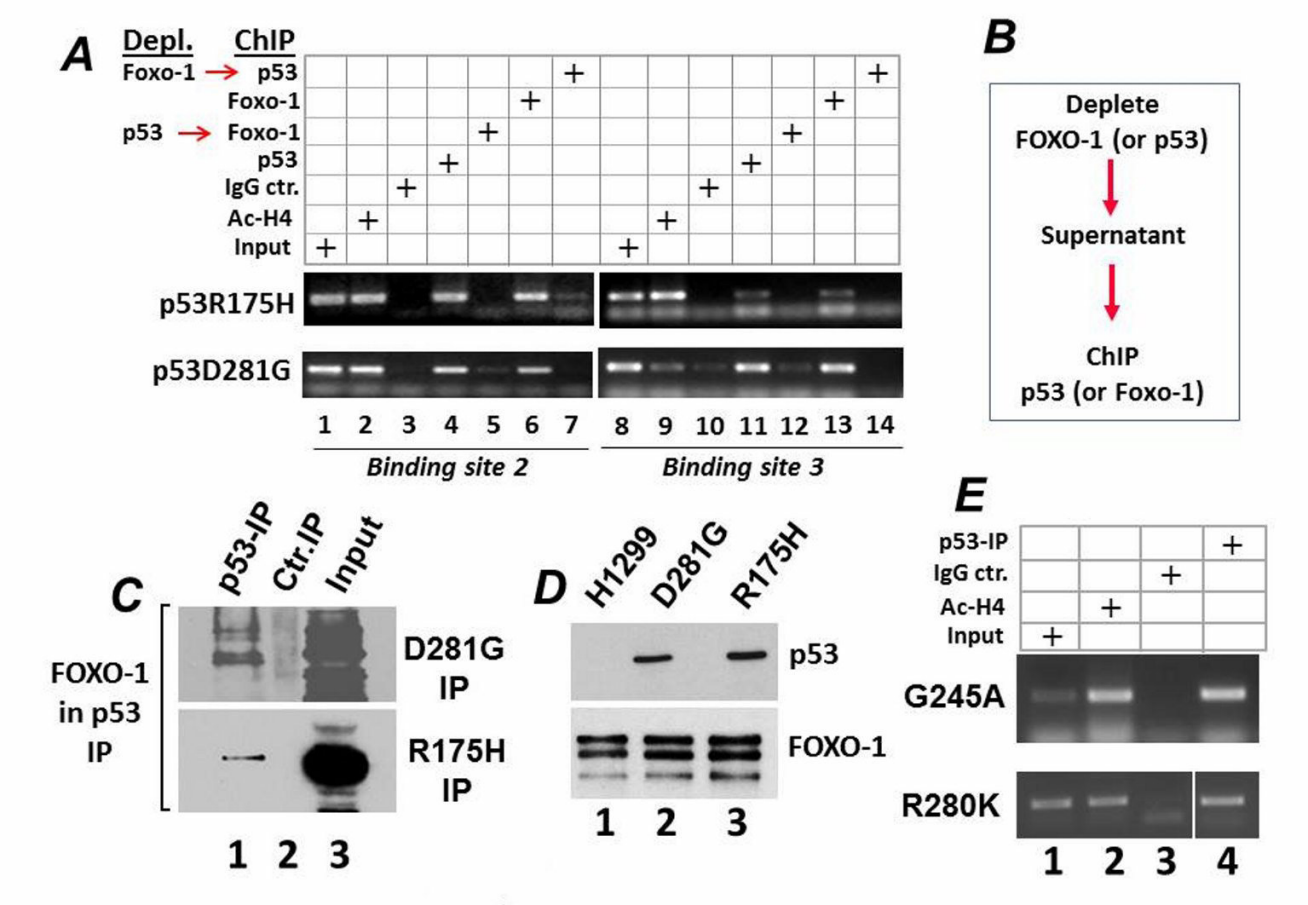
A. Chromatin immuno-precipitation assays performed on the Binding Site 2 and 3 of the CIC promoter. The procedure for the immuno-depletion is schematically illustrated in B. Cell extracts were subjected to a first immuno-precipitation with the anti-p53 or anti-FOXO-1 antibody, and the supernatant of these reactions was used for the reciprocal ChIP with the anti-FOXO-1 or anti-p53 antibodies, respectively. In panel A cells expressing p53R175H or p53D281G were immuno-precipitated with antibodies directed against acetylated histone H4 (lanes 2 and 9), or with IgG control (lanes 3,10), or with anti-p53 DO1 antibody (lanes 4,11), or anti-FOXO-1 antibody (lanes 6,13). The supernatant of the cell extracts subjected to the first immuno-precipitation with the anti-p53 or anti-FOXO antibodies were then immuno-precipitated with the anti-FOXO-1 (lanes 7, 14), or anti-p53 (lanes 5 and 12) antibodies. Lane 1 contains input levels. C. Nuclear extracts derived from H1299 cells expressing p53R175H or p53D281G, as indicated at the right of each panel, were prepared and immuno-precipitated either with a control IgG antibody (lane 2), or with the anti-p53 antibody DO1 (lane 3). Lane 1 contains 1/50 of the total extracts, probed for FOXO-1. D. In parallel experiments the levels of p53 (upper panel) or FOXO-1 were assessed in the cell lines indicated at the top of the panel. E. ChIP assays were performed in cell extracts derived from cells expressing p53G245A (top panel), or from MDA-231 cells expressing p53R280K (bottom panel).

This result prompted us to interrogate the DNA binding ability of mutant p53 with different regions of the CIC promoter relative to the FOXO-1 binding sites. To this end, we employed chromatin immuno-precipitation assays (ChIP). There are several binding sites for FOXO family members some of which partially overlap with binding elements for other Forkhead transcription factors, including Hepatocyte Nuclear Factor 3-alpha (HNF-3A, or FOXA1 [28]), (Supplemental Figure S3). By dividing the CIC promoter into 5 fragments each encompassing approximately 300 base pairs, we found that that both p53R175H and p53D281G interacted strongly with two different regions of the CIC promoter (designated Binding Site 2 and 3, or BS2 and BS3 in Figure 3A; Figure 4A, lanes 4 and 11). To next determine whether mutant p53 is recruited to these sites through an interaction with FOXO-1, we performed immuno-depletion experiments followed by ChIP assays (strategy depicted in Figure 4B). Cell extracts containing chromatin-bound proteins were first subjected to an immuno-precipitation with either the anti-p53 (lanes 5, 12) or anti-Foxo1 (lane 7,14) antibodies. The supernatants derived from these reactions were then re-immuno-precipitated with the anti-FOXO or anti-p53 antibodies and subjected to PCR assays. As shown in Figure 4A depletion of FOXO-1, impaired the occupancy of both the p53R175H and the p53D281G proteins on the CIC promoter (compare lanes 4 and 11 with lanes 7 and 14, respectively) and, conversely, depletion of p53 compromised FOXO-1 binding (compare lanes 6 and 13 with lanes 5 and 12). Furthermore, although neither of these mutants affected FOXO-1 expression levels, FOXO-1 could be detected in soluble complexes with either p53R175H or p53D281G in immuno-precipitation experiments (Figure 4CD). Importantly, other mutants, including p53G245A and p53R280K similarly bound to the CIC promoter (Figure 4E). Taken together with the luciferase assays shown in Figure 3D, these results thus identify a novel interaction of p53 mutants with the transcription factor FOXO-1, which is responsible for the recruitment of these proteins to the CIC promoter and for the induction of CIC transcription.

### CIC contributes to the gain of oncogenic function of p53 mutants and its inhibition enhances chemo-sensitivity to cisplatin

**Figure 5:**
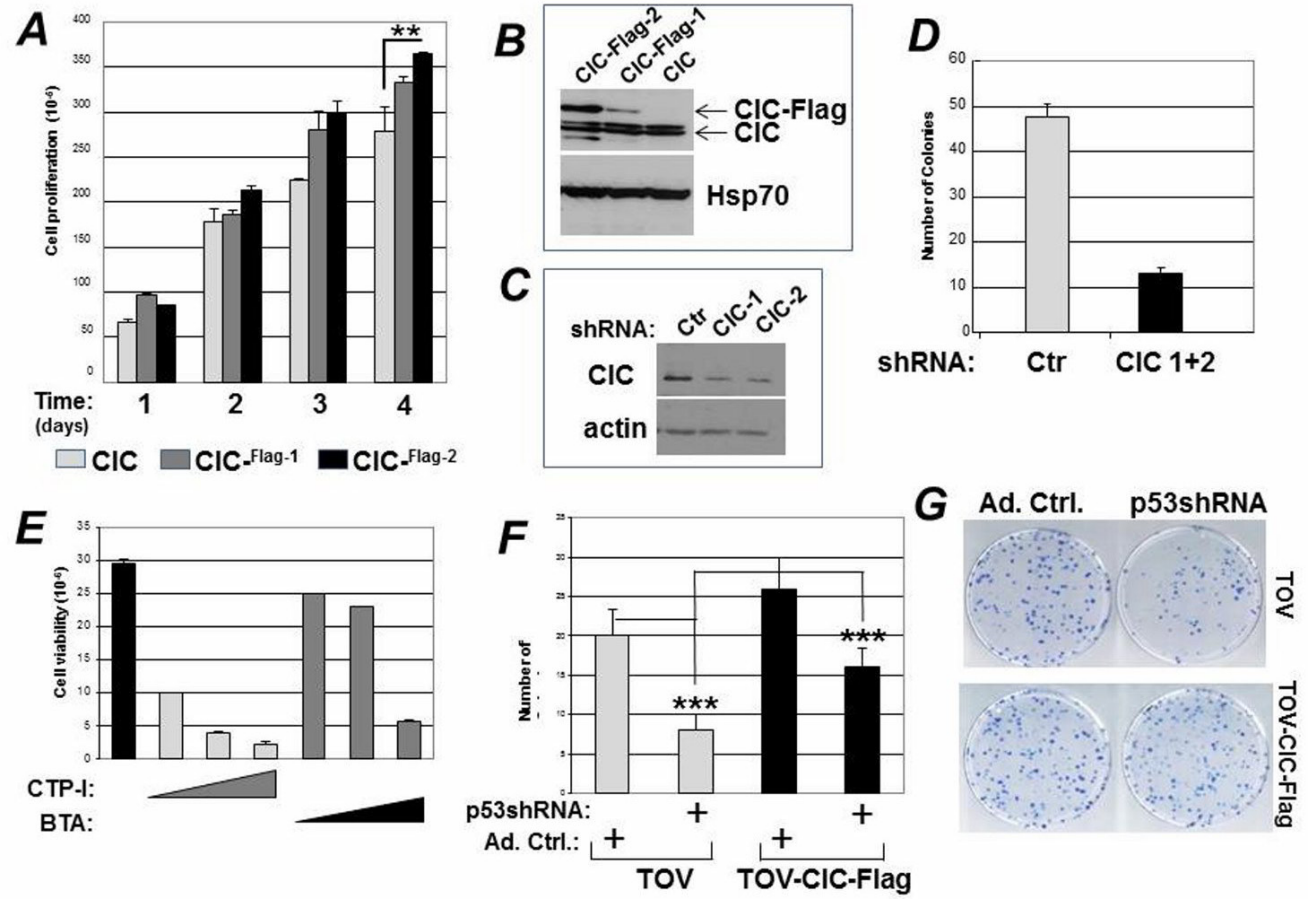
CIC affects the GOF activity of mutant p53 A. Naive TOV cells expressing endogenous CIC, or TOV multi-clonal populations harboring epitope-tagged CIC (CIC-Flag-1, CIC-Flag-2) were plated at 500 cells/well and their proliferative capacity was studied for four days. B. Immuno-blot showing the expression levels of endogenous CIC and the CIC-Flag clones in TOV cells. C. Expression levels of CIC in the presence of control shRNA, or of two specific CIC shRNAs. D. Colony forming assays in TOV cells transfected with the control shRNA, or with the CIC specific shRNA after one week of selection with puromycin. E. Cell viability assessed with trypan blue exclusion of H1299 cells treated with 0.1 mM, 0.5 mM or 1 mM of CTP-I or BTA. F-G. Naive TOV cells or TOV cells expressing CIC-Flag-2 were infected with control adenovirus, or with adenovirus harboring the specific p53 shRNA. Cells were plated and their colony forming ability was assessed.

Many p53 mutants acquire neomorphic gain of oncogenic function (GOF) activities, through which they promote proliferation. Therefore, we next investigated whether CIC promotes mutant p53 GOF. We previously showed that inhibition of CIC with specific shRNAs or with the specific CIC inhibitor compound benzene-tricarboxylate (BTA), hampers the oncogenic potential of MDA-231 cells, which express the GOF mutant p53R280K [14]. We then expanded these experiments to explore the effects of CIC in other cell lines harboring p53 mutations. First, we used ovarian cancer cells TOV, which express the hot spot p53R175H mutant, to construct TOV derivative clones harboring the cDNA expressing epitope-tagged Flag-CIC. After antibiotic selection, multi-clonal cell populations were pooled together and we studied the proliferation rates of two clones differing in CIC expression levels (indicated as CIC-Flag-1 and CIC-Flag-2, Figure 5AB). Over-expression of CIC led to a modest, but statistically significant, dose-dependent enhancement of proliferation rates (Figure 5A). Conversely, the co-expression of two previously validated CIC shRNAs [14], led to a reduction of CIC levels (Figure 5C) and hampered proliferation (Figure 5D). Two different chemical inhibitors of CIC, BTA and 4-Chloro-3-[[(3-nitrophenyl)amino]sulfonyl]-benzoic acid, CNFASB or CTP-I [29], also dramatically reduced proliferation rates (Figure 5E).

To next explore in further depth the effects of the CIC protein on mutant p53 GOF properties, TOV cells or the TOV cells over-expressing CIC, were infected with either an adenovirus expressing the p53 shRNA or with a control adenovirus, and cell proliferation was assessed using colony forming assays. In naive TOV cells the p53 shRNA reduced colony forming ability, consistent with the well-documented GOF properties of p53R175H [4-6]. However, colony forming rates were nearly entirely rescued in the TOV cell line over-expressing CIC (Figure 5FG). Thus, these findings indicate that the proliferation advantage conferred by mutant p53 relies at least in part, upon CIC, as CIC over-expression overcomes the effects of the p53 mutant knockdown.

One of the most challenging problems faced in the treatment of *ovarian cancer*, as well as of other solid tumors is the development of resistance to platinum-derived agents, which are often used as first line therapeutics. The presence of p53 mutations is a well known contributor to drug resistance [30, 31]. Recent genome-wide transcriptional analyses of tumors that are either chemo-sensitive or chemo-resistant to platinum or carboplatin treatment have identified specific genetic signatures linked to chemo-resistance [32,33]. The analysis of these data, extracted from the geoprofile database, showed that carboplatin resistant tumors derived from patients or from cell lines display increased expression levels of CIC (Supplemental Figure S4A). To then determine whether CIC affects the sensitivity to platinum compounds, TOV cells were treated with different doses of cisplatin in the presence or absence of BTA. As anticipated, treatment with BTA displayed a chemo-sensitizing effect (Figure S4B), leading to a reduction of the IC50 of cisplatin from 150 nM to 37 nM. Since we have previously shown that systemic BTA treatment is well tolerated, at least in the adult mouse [14], the data imply that co-treatment with CIC inhibitors may re-sensitize certain resistant tumors to cisplatin or may permit achieving clinically effective tumor killing while employing more tolerable, less toxic doses of cisplatin.

### Inhibition of CIC activity blunts mutant p53 GOF activity in vivo

**Figure 6:**
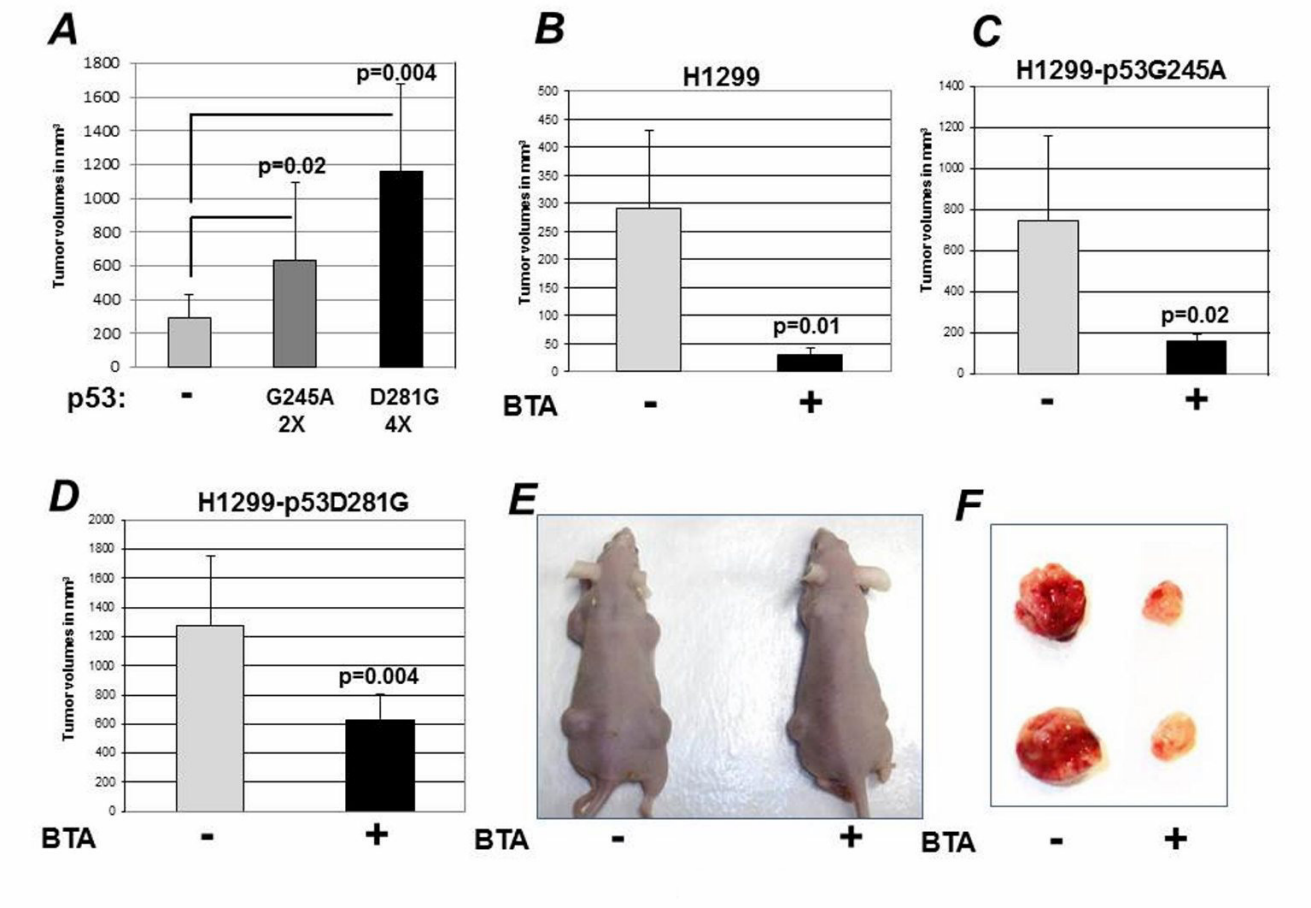
Inhibition of CIC blunts the GOF activity of mutant p53 A. Naive H1299 cells, or H1299 cells expressing p53G245A or p53D281G mutant were injected in the flank of nude mice. Tumor volumes were assessed several weeks after implantation. B-D. Tumor volumes assessed after implantation in nude mice of naive H1299 cells or H1299 cells expressing p53G245A or p53D281G either mock treated (−) or BTA treated (+). Bars represent standard deviations; p-values between different groups are shown in all panels. E-F. Representative mice (E) or tumors (F) derived from these experiments are shown.

To further explore the relationship between mutant p53 and CIC, we next employed a syngeneic system for studying p53 function by using the previously described p53-null lung cancer cells H1299, engineered to express either p53D281G or p53G245A [24,25]. To test the hypothesis that CIC inhibition blunts the oncogenic activity of mutant p53, we performed tumor transplantation experiments in nude mice. As shown in Figure 6A, and consistent with GOF activities, the expression of p53G245A or p53D281G significantly enhanced the tumorigenicity of H1299 cells (Figure 6A). Treatment with BTA nearly completely abrogated tumor formation in the p53-null H1299 background, as we have previously shown [14]. In addition, BTA significantly reduced tumor size in the case of both p53G245A and p53D281G (Figure 6B-E). Importantly, tumors that arose in BTA treated mice displayed not only a reduction in size, but were also significantly hypo-vascular (Figure 6F), suggesting that CIC inhibition might interfere with angiogenesis and/or with the tumor-stroma interaction. It should be noticed that tumor inhibition induced by BTA was more prominent in naive H1299-p53 null-derived tumors, relative to tumors harboring p53D281G or p53G245A. In keeping with the higher CIC levels in cells harboring p53 mutations compared to p53 null cells (Figure 2), this results suggests that higher doses of BTA may be needed to achieve complete CIC inhibition in p53 mutant tumors.

### Elevated CIC levels predict poor survival in lung cancer

**Figure 7:**
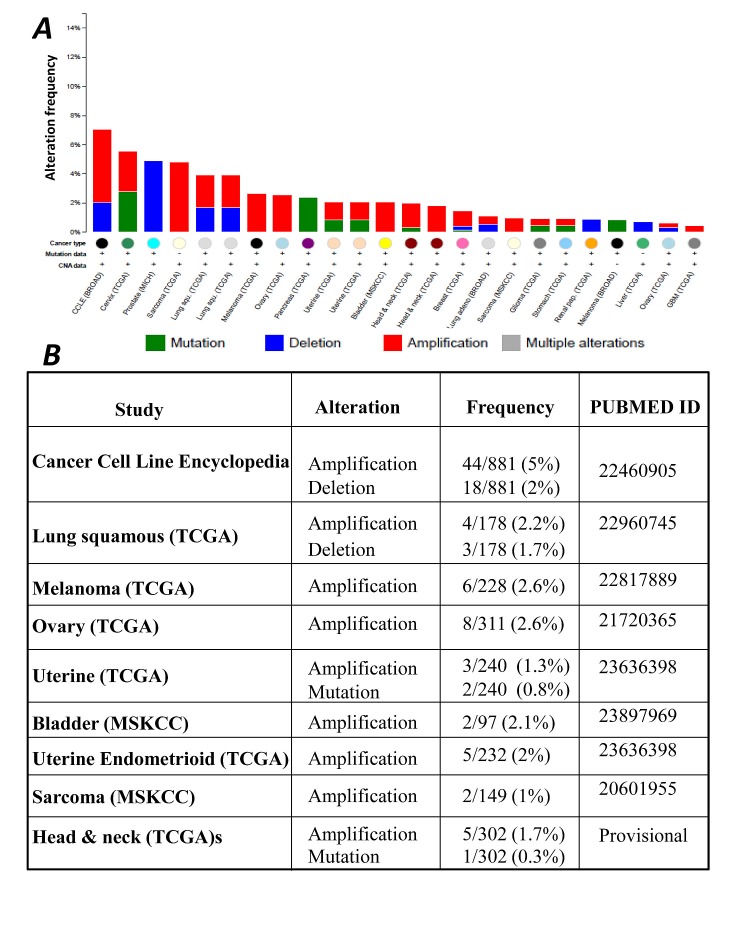
A. Spectrum of genomic alterations of SLC25A1 (CIC) across different human tumor types and cancer cell lines extracted from the cBioPortal database. B. The study, the type of alteration(s) and the pubmed ID reporting CIC alterations are summarized in the table.

**Figure 8:**
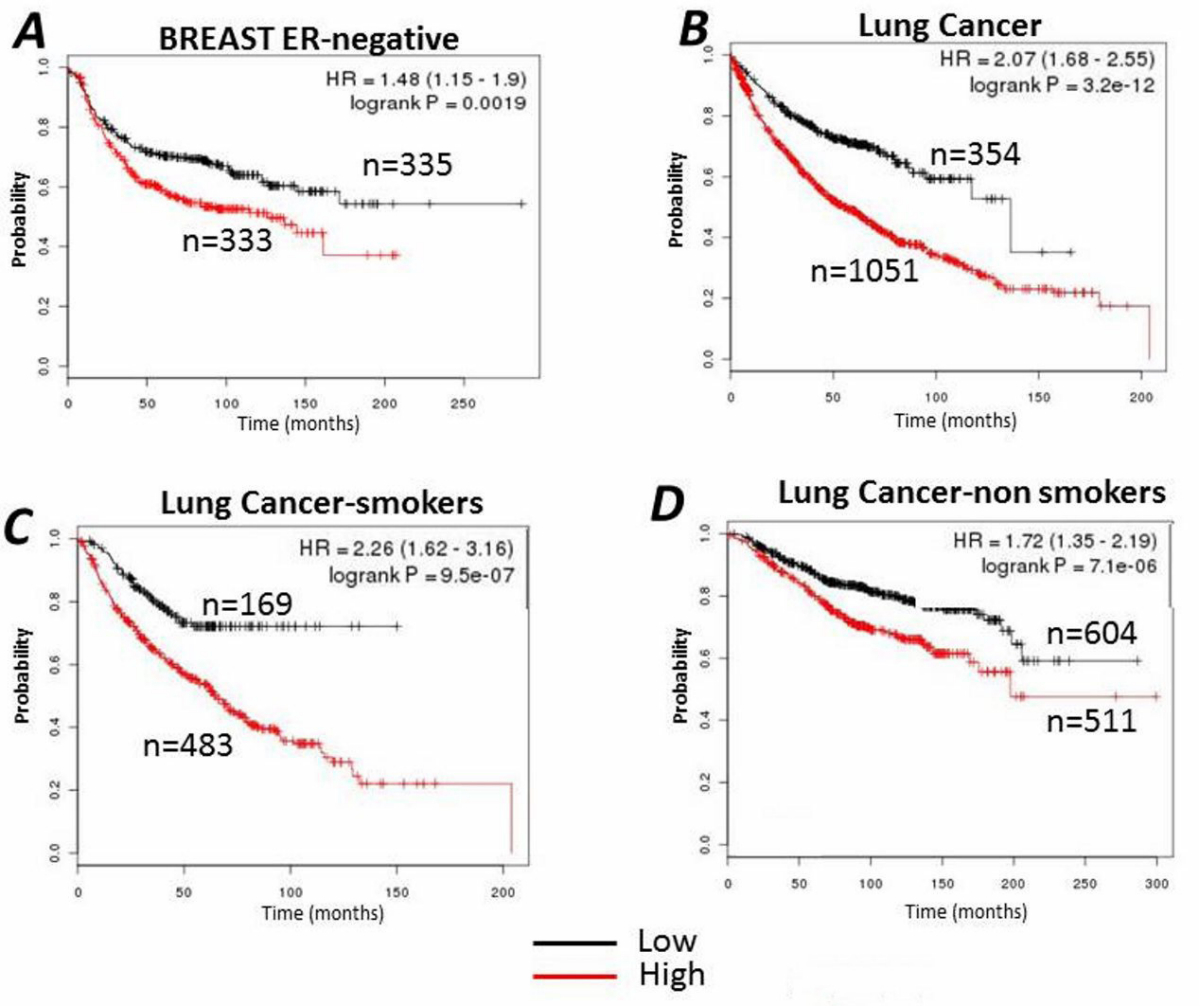
Kaplan–Meier survival curves relative to *SLC25A1* (CIC) expression were generated for breast cancer (A), or lung cancer (B) Data were analyzed with the KM-plotter (http://kmplot.com/analysis/). In the case of lung cancer, the cohort of patients was divided into “smokers” (C) or “non smokers” and by allowing the software to select the best cut-off. Red and black lines indicate patients with higher and lower CIC expression respectively. The total number of patients in the two categories are shown. Hazard ratios (HR) and *p* values (log rank *p*) are shown at the top of the panel.

To further extend the significance of our findings, we analyzed available databases in order to correlate CIC expression with clinically relevant parameters. We have previously shown that elevated CIC expression levels are detected in several tumor types and cancer cell lines [14]. Our analysis of the CBioPortal database for cancer genomics also demonstrated alterations in copy number as well as mutations of the CIC gene in various tumors, including lung, ovary, bladder and head and neck cancers (Figure 7). Among the copy number alterations identified, amplifications were more frequent relative to deletions. Importantly, CIC is a validated amplified gene in lung squamous cell carcinoma [http://igdb.nsclc.ibms.sinica.edu.tw/significant_genes.php], and it is up-regulated in lung adenocarcinomas as well (see [14] and Figure 8 below].

We next employed the Kaplan–Meier software (KM-plotter) that has the capability to assess the effect of 22,277 genes on survival in 4,142 breast, 1,715 *lung cancers* and 1,464 ovarian cancers [34-36]. In the case of breast cancer, the most significant negative correlation between high CIC levels and survival rates was seen in Estrogen-Receptor Negative (ER-) breast cancers (Figure 8A). In the case of lung cancer, the ability of high expression levels of CIC to predict survival was quite remarkable (Figure 8B). First, the analysis of these datasets confirmed that a large portion of patients affected by lung cancer displays high CIC expression levels. Second, the overall survival of the patients with high CIC levels was dramatically reduced, compared to patients with low CIC expression. Given that lung cancer and the occurrence of p53 mutations are strongly associated with cigarette smoking, we next evaluated the performance of CIC as a marker of survival in smoker versus non-smoker patient populations. As shown in Figure 8CD the expression levels of CIC generated a negative prognostic stratification which was much more significant in smokers than in non-smokers. The implications of these analyses is that patients with low CIC levels have a much higher probability to survive lung cancer, with a remarkable overall difference in survival of approximately 5 years.

## DISCUSSION

Our results demonstrate that CIC is an important target for cancer therapy and provide a strong rationale for the employment of its inhibitors for the treatment of tumors harboring p53 mutations. Furthermore the data establish a strong rationale for the assessment of *CIC* expression in lung cancer, given that CIC appears to have negative prognostic significance in these tumors.

The frequency of p53 mutations is particularly high in lung, ovarian and triple receptor negative breast cancers [31,37, 38], paralleling high expression levels of CIC in these tumors [14]. There is evidence that mutp53 proteins acquire the capability to promote tumorigenesis by implementing a transcriptional program that is distinct from that elicited by the wild-type protein. Consistent with this view, mutant but not wild-type p53 was able to enhance the transcription of the CIC promoter. Moreover, by interrogating available databases, we have found that higher expression of the CIC mRNA is detected in primary tumors harboring p53 mutations. This result is consistent with our studies performed in cancer cells in culture, where we found a strong correlation between high CIC levels and the presence of mutant p53. Taken together with results demonstrating that inhibition of CIC blunts tumorigenesis in p53 mutant cancers, our study indicates that CIC is an important component of the program by which mutants of p53 gain oncogenic activity. Importantly, in our experimental conditions wild-type p53 was unable to activate transcription of the CIC promoter, while we consistently observed a modest but reproducible transcriptional repression. Since CIC is highly expressed in the adipose tissue where it likely regulates lipid synthesis, our results are apparently consistent with previous reports showing that the expression of other enzymes involved in lipid synthesis and metabolism are repressed by wild-type p53 [9, 39,40].

Our previous data demonstrated that CIC promotes oncogenesis with complex molecular mechanisms. An important activity of CIC consists in promoting the export of citrate from the mitochondria to the cytoplasm. Recent work has shown that various p53 mutants with GOF properties stimulate sterol and lipid biosynthesis and activate the mevalonate pathway, an activity through which these mutants subvert the normal architecture of the mammary gland [9]. Since cytoplasmic citrate is the only source for sterol and fatty acid synthesis it is very likely that CIC is involved in this activity of mutant p53. *De novo* lipid synthesis has also been implicated in the acquisition of an invasive and metastatic phenotype, which is similarly promoted by certain p53 mutants [3,9,41,]. Furthermore, our mechanistic studies showed that alterations in mitochondrial activity and turnover are primarily responsible for the anti-tumor effects of CIC inhibitors [14]. Indeed, the molecular signatures of cells where CIC activity is impaired consist of a reduction of mitochondria mass, production of Reactive Oxygen Species (ROS) and induction of mitochondrial autophagy, also called mitophagy, which is in turn responsible for loss of viability due to CIC inhibition [14]. Our data also showed that CIC bypasses the glycolytic addiction of tumors and promotes OXOPHOS and ATP production, ultimately enacting survival in response to glucose starvation or to mitochondrial respiration injury. These two forms of stress essentially recapitulate nutritional and oxidative stress signals that all ensuing tumors face and must overcome due to the inadequacy and irregularity of the vasculature. More recently, mitochondrial alterations similar to those described by our group were reported in patients harboring germ-line inactivating CIC mutations [42,43]. Thus, it has now been proposed that CIC be classified as a gene involved in mitochondrial diseases [42,43]. In this respect it is important to note that while it is very clear that wild-type p53 affects mitochondrial function, respiratory activity and glycolysis [44,45], it is currently unknown how p53 mutants affect these activities and the cancer promoting metabolic program. Therefore the finding that CIC is a transcriptional target of at least some types of p53 mutants provides a potentially important link between these proteins and the metabolism of tumor cells. The clarification of how the mutp53-CIC crosstalk influences these important activities will be an object of future studies. An additional important finding of this study consists in the contribution of the transcription factor FOXO-1 to the ability of mutant p53 to regulate CIC transcription. Although the available data indicate that FOXO-1 might function as an anti-oncogene, this transcription factor plays a key role in metabolic adaptation to starvation, in regulation of lipid catabolism during stress as well as in autophagy [46,47]. Therefore, it is attractive to speculate that the cross-talk of mutant p53 with FOXO-1, and possibly other FOXO-1-dependent transcriptional targets, might promote survival of p53 mutant cells in the nutrient and oxygen-deprived tumor microenvironment. However, we cannot exclude that other transcription factors or other members of the *Forkhead* box family also contributes to mutp53-dependent regulation of CIC transcription.

Viewed together our data indicate for the first time that CIC is a relevant target of GOF mutp53 proteins, demonstrate that assessment of *CIC* expression might have clinical relevance for predicting survival of patients harboring p53 mutant tumors and further, they imply that the employment of CIC inhibitors may improve chemo-sensitivity.

## METHODS

### Cell lines and Reagents

The TOV cell lines employed in this study were obtained from the tissue culture core facility at LCCC and the H1299 cells were obtained from ATCC. The H1299 constitutively expressing the p53D281 were a kind gift from Dr. Prives and the cells expressing p53G245A have been described previously [24,25]. The MDA-231shp53 and MDA-468shp53 were obtained from Dr. Jill Bargonetti and were described previously [9]. All the cells were grown in Dulbecco' s modified Eagle's medium (DMEM, 25 mM glucose, with glutamine and pyruvate from Invitrogen) and supplemented with 10% fetal calf serum (FCS). The CIC specific shRNA vectors were purchased from Origene (#TG316728 and #TR316728, untagged or GFP-tagged). The vectors expressing human CIC untagged or Flag-Myc epitope tagged were also from Origene (#SC120727 and RC200657, respectively). The antibodies used in this study were as follows. The anti-CIC antibody from Santa Cruz Biotech, # sc-86392 employed at 1:1000 dilution in immuno-blot. The anti-p53 antibodies were the FL393 (Santa Cruz) or the DO1 Ab (Santa Cruz or Life Technologies). The FOXO-1 polyclonal antibody and shRNA were from Santa Cruz.

### Chromatin Immuno-Precipitation Assays

These were performed as previously described. Briefly, the cells were cross-linked with 1% (w/v) formaldehyde-PBS solutions for 10 min at room temperature, formaldehyde was then inactivated by the addition of 125 mM glycine. Chromatin extracts were sonicated to obtain DNA fragments with size of 300-800 bp and then they were immunoprecipitated overnight with rotation using the anti-p53 antibody (DO1,Santacruz Biotechnology) or the anti-Foxo1 antibody (Santa Cruz Biotechnology). On the following day, protein A/G magnetic beads(Millipore) that had been previously blocked with salmon sperm DNA were added to each reaction to precipitate antibody-DNA-protein complexes. The precipitated complexes were then separated using magnetic separator to separate immuno-precipitated complex and supernatant. The immuno-precipitated complex was then washed and incubated at 62°C overnight in parallel with ‘input’ samples to reverse the crosslinking of DNA. The DNA was then separated from the complex using a magnetic separator (Invitrogen), the DNA was purified using Qiagen-PCR purification kit prior to its use in the PCR reaction. For the immuno-depletion experiments the soup obtained after the ChIP with the first Ab, was incubated with anti-FOXO-1 or anti-p53 antibodies and subjected to a second ChIP. The precipitated DNA was subjected to PCR reactions for 30–35 cycles as previously described.

### Primers used for the ChIP assays

The primers used for amplification of Binding Sites 1 and 2 were as follows: BS2: CIC promoter forward: 5' g c C A g g t t c t c t g g c t g a c 3'; CIC promoter reverse: 5' G c T G g a g T G a c a T G c t c c T T 3'. For BS3: CIC promoter forward: 5' A A t g g g a g g C A g g g a C A c 3'; CIC promoter reverse: 5' c c a a g a g g c T G a g a g t c c T T T 3'.

### Immunoprecipitations and immunoblots

Preparation of cell extracts and immuno-precipitations were performed as previously described [14,24,25]. For assessment of total CIC levels cell extracts were prepared in RIPA buffer [14], for immuno-precipitation experiments we used Buffer A (20% Glycerol; 40 mM Tris HCl pH 7.9; 0.4 mM EDTA; 0.2% Tween 20; 100 mM KCl), supplemented with protease inhibitors, 5 mM DTT or Betamercaptoethanol, 10 mM N-ethylmalemide (NEM), as well as with TSA (500 μM). Protein extracts were combined with the indicated antibodies, precipitated with immobilized protein A beads (Pierce), and subjected to SDS-PAGE, followed by transfering to PVDF membranes (Milipore). Chemiluminescence was performed with the WestPico system (Pierce).

### Reverse transcription polymerase chain reaction (RT-PCR)

For detection of CIC mRNA, total RNA was extracted from cells using a commercially available kit (Quiagen), followed by Reverse transcriptase PCR (SuperScript III first-strand synthesis system, Invitrogen), which was performed with random hexamer primers and 2 mg of RNA to obtain sufficient cDNA for analysis. The primers were: forward 5'Catcgagatctgcatcacct3'; reverse: 5'Caaccccaacaagcccatgaa3'.

### Luciferase Assays

The full length CIC promoter cloned upstream of the PGL-3 vector and its corresponding SREBP-1 mutant, were previously described. One g of the luciferase reporter was transfected as indicated in the Figure legend with a Calcium Phosphate Protocol (Promega), together with the indicated concentrations of the plasmids expressing wild-type or mutant p53. Luciferase activity was monitored 24 hours after with an available kit (Promega). Data from duplicate or triplicate experiments were plotted in excel spread sheet and analyzed.

### Cellular proliferation and colony forming assays

The proliferative capacity of cells was assessed by plating cells in a duplicate or triplicate. Treatments were applied as indicated in the Legend of the Figure and viable or dead cells were measured after 48-72 hours with trypan blue exclusion by emoploying the Countess®Automated Cell Counter (Life Tech.), according to the manufacturer instruction. For colony forming assays cells were plated in either 6 or 10 cm tissue culture dishes at a concentration of 500 or 1000/dish, respectively. Colony forming ability was assessed 4-10 days. Colonies were fixed with 4% *paraformaldehyde* and stained with a solution containing 0.5% crystal violet.

### Mice and tumors

To produce tumor xenografts 5 × 10^6^ cells were resuspended in PBS and injected subcutaneously in the flanks of nude mice (Taconics). Mice were randomized to receive either PBS or a PBS solution of BTA at a concentration 26 mg/kg which was administered via intra-peritoneal route three times a week. Mice were pre-treated twice prior to the inoculation of tumor cell lines. Once detectable tumors started to form, their size was measured with a caliper in three dimensions. Serial measurements were made at two-three day intervals after the identification of the initial cellular mass to determine growth curves *in vivo*. Tumor volumes were calculated using the formula for a prolate ellipsoid *V*_T_ = π/6 · *L* · *W* · *D*, where *L* is length, *W* is width, and *D* is depth. All animal studies were approved by the Georgetown University Institutional Animal Care and Use Committee.

### Analysis of SLC25 alterations in human patient samples

We analyzed the Oncomine database (https://www.oncomine.org) or the cBioPortal database (http://www.cbioportal.org) for Cancer genomics as previously described by others [e.g., 48]. To simultaneously monitor genomic alterations of SLC25A1 (CIC) as well as changes in its expression levels in p53 mutant tumors (e.g., Figure S1), the gene names SLC25A1 and tp53 were provided as input in the query form and each specific tumor type generated in the Oncoplot was individually analyzed for SLC25A1 expression and p53 mutations. The analysis of Kaplan-Meier survival analysis was conducted with the KM plotter, by entering SLC25A1 as the input and allowing the software to select the best cut-off.

### Statistical analysis

Data are expressed as means ± standard deviations (SD). The two-tailed Student *t* test was used for all statistical analysis of experiments presented and Excel was used for statistical calculations. Significant differences are indicated using the standard Michelin Guide scale (*P* < 0.05, significant; *P* < 0.01, highly significant; *P* < 0.001, extremely significant).

## SUPPLEMENTARY FIGURES


