# Supplementary material for: SLC25A1, or CIC, is a novel transcriptional target of mutant p53 and a negative tumor prognostic marker
Source: Oncotarget. 2014 Mar 16;5(5):1212–25. doi: 10.18632/oncotarget.1831 (PMC4012738; doi:10.18632/oncotarget.1831)
Supplement: Supplementary file 1 [file oncotarget-05-1212-s001.doc]

Supplementary file for Kolukula et al.


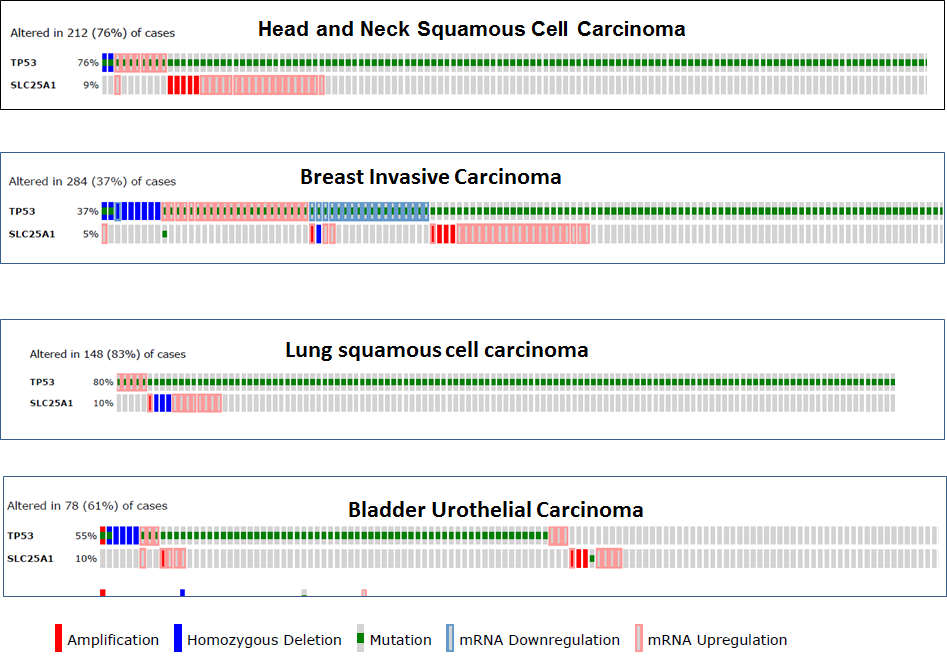


Supplementary Figure S1. Co-occurrence of alterations of CIC (SLC25A1) in p53 mutant tumors. The OncoPrint maps were generated with the cbBioPortal database and show the frequency of SLC25A1 (CIC) expression levels, or genetic alterations occurring concomitantly with the presence of p53 mutations in the tumor types indicated at the top of each panel. The individual genes are represented as rows and individual patients are represented as columns. Each column corresponds to a different patient.

Supplementary Figure S2. Comparison of CIC alterations with known targets of p53 mutants. In this case OncoPrint maps were generated to compare the expression levels of SLC25A1 (CIC), with the expression levels of cyclin A1 (CCN1), cdk1, 3-Hydroxy-3-Methylglutaryl-Coenzyme A Reductase (HMGCR) and Mevalonate (Diphospho- Decarboxylase (MVD).

The

| Name | Sequence | Position (0-based) | Strand | Score | p-value |  |
| --- | --- | --- | --- | --- | --- | --- |
| [FOXO1 (M00473)](http://biogrid-head.engr.uconn.edu/lasagna_search/transfac_matrices/M00473.html) | AACACG | 896 | + | 7.24 | 0.0054 |  |
| [FOXO1 (M00473)](http://biogrid-head.engr.uconn.edu/lasagna_search/transfac_matrices/M00473.html) | AAACAAG | 903 | - | 7.17 | 0.0066 |  |
| [FOXO1 (M00473)](http://biogrid-head.engr.uconn.edu/lasagna_search/transfac_matrices/M00473.html) | AACAAG | 1149 | - | 6.96 | 0.00955 |  |
| [FOXO1 (M00473)](http://biogrid-head.engr.uconn.edu/lasagna_search/transfac_matrices/M00473.html) | ACAGG | 653 | + | 6.76 | 0.013475 |  |
| [FOXO1 (M00473)](http://biogrid-head.engr.uconn.edu/lasagna_search/transfac_matrices/M00473.html) | ACACG | 897 | + | 6.6 | 0.017625 |  |
| [FOXO1 (M00473)](http://biogrid-head.engr.uconn.edu/lasagna_search/transfac_matrices/M00473.html) | ACACG | 576 | - | 6.6 | 0.017625 |  |
| [FOXO1 (M00473)](http://biogrid-head.engr.uconn.edu/lasagna_search/transfac_matrices/M00473.html) | ACAAG | 1099 | + | 6.32 | 0.0281 |  |
| [FOXO1 (M00473)](http://biogrid-head.engr.uconn.edu/lasagna_search/transfac_matrices/M00473.html) | CAGG | 827 | + | 6.12 | 0.0346 |  |
| [FOXO1 (M00473)](http://biogrid-head.engr.uconn.edu/lasagna_search/transfac_matrices/M00473.html) | CAGG | 401 | - | 6.12 | 0.0346 |  |
| [FOXO1 (M00473)](http://biogrid-head.engr.uconn.edu/lasagna_search/transfac_matrices/M00473.html) | CAGG | 838 | - | 6.12 | 0.0346 |  |
| [FOXO1 (M00474)](http://biogrid-head.engr.uconn.edu/lasagna_search/transfac_matrices/M00474.html) | AAGTTGTACAC | 562 | - | 10.24 | 0.005675 |  |
| [FOXO1 (M00474)](http://biogrid-head.engr.uconn.edu/lasagna_search/transfac_matrices/M00474.html) | AGTTTGAAGAC | 879 | + | 8.11 | 0.015575 |  |
| [FOXO1 (M00474)](http://biogrid-head.engr.uconn.edu/lasagna_search/transfac_matrices/M00474.html) | AAGTTGTACACT | 561 | - | 8.1 | 0.015625 |  |
| [FOXO1 (M00474)](http://biogrid-head.engr.uconn.edu/lasagna_search/transfac_matrices/M00474.html) | CGGCTGTATTC | 1114 | - | 6.77 | 0.026325 |  |
| [FOXO1 (M00474)](http://biogrid-head.engr.uconn.edu/lasagna_search/transfac_matrices/M00474.html) | AAGTTGTACACTG | 560 | - | 6.49 | 0.0297 |  |
| [FOXO1 (M00474)](http://biogrid-head.engr.uconn.edu/lasagna_search/transfac_matrices/M00474.html) | CACGTGTTTTC | 1327 | + | 6.42 | 0.03015 |  |
| [FOXO1 (M00474)](http://biogrid-head.engr.uconn.edu/lasagna_search/transfac_matrices/M00474.html) | GCTGTATTCTCC | 1111 | - | 6.03 | 0.03565 |  |

Supplementary Figure S3. A. Structure of the CIC promoter and the position of the FOXO-1 binding sites. B. The specific position of the FOXO-1 binding sites detected with the *LASAGNA* software is summarized in the table.

Supplementary Figure S4. A. Relative mRNA expression levels of CIC in patients affected by ovarian cancer and stratified in carboplatin sensitive and carboplatin resistant (see also text for explanation). Data were extracted from the Geoprofile database and analyzed. B. TOV cells were first treated with the indicated concentrations of cisplatin for 16 hours, followed by removal of cisplatin and incubation of the cells in the absence (gray bars) or presence (black bars) of 500 μM BTA.
